# Identification of loci involved in childhood visual acuity and associations with cognitive skills and educational attainment

**DOI:** 10.1038/s41539-023-00175-w

**Published:** 2023-07-25

**Authors:** Judith Schmitz, Filippo Abbondanza, Krzysztof Marianski, Michelle Luciano, Silvia Paracchini

**Affiliations:** 1 School of Medicine, University of St Andrews, St Andrews, Scotland, UK; 2 Biological Personality Psychology, Georg-August-University Goettingen, Goettingen, Germany; 3 Department of Psychology, The University of Edinburgh, Edinburgh, Scotland UK

**Keywords:** Human behaviour, Education, Object vision

## Abstract

Visual acuity significantly contributes to quality of life. Deficits in childhood are associated with reading difficulties, which can have detrimental effects on education outcomes. In adults, it has been observed that vision defects such as myopia are associated with higher educational attainment (EA). Understanding genetic factors contributing to visual acuity could help to dissect its links with cognitive skills, neurodevelopmental conditions, and education. We examined associations between distance visual acuity, cognitive measures including school grades, and neurodevelopmental conditions in a longitudinal cohort of British children (ALSPAC, *n* = 6807, M age = 11.8). We performed a genome-wide association study (GWAS, *n* = 5571) on visual acuity and tested for genetic associations with relevant phenotypes using polygenic scores (PGS) and genetic correlation analyses. Visual acuity was associated with better cognitive performance and school grades, and reduced in individuals with reading difficulties compared to controls. GWAS revealed genetic associations at the *NPLOC4* locus and highlighted other genes involved in sensory function. In line with positive genetic correlations between visual acuity and cognitive measures, EA PGS were positively associated with visual acuity, while there was a less robust negative association with myopia PGS. In conclusion, increased visual acuity is associated with a range of positive outcomes, including better school grades. Our results suggest an association between a higher EA PGS and slightly increased visual acuity in childhood. This could indicate gene-environment correlation, in which environmental exposures linked to higher EA might have detrimental effects on vision offsetting the initial positive effect.

## Introduction

Visual function has a significant impact on perceived quality of life, which is expressed vividly in the finding that on average, elderly subjects with reduced visual function (*n* = 325) would trade 20% to 50% of remaining lifetime for perfect vision^1^. Visual acuity is defined as the visual system’s ability to resolve spatial detail and usually measured as distance visual acuity with the chart placed 20 feet or 6 metres away. The most common defect underlying reduced visual acuity is uncorrected refractive error. Refractive error is present when the light from an object of interest is not focussed accurately onto the retina and most commonly due to the eyeball being longer or shorter than normal^2^. In children, the risk for significant uncorrected myopia (typically defined as ≤ -8 to -5 dioptres) is inversely related to visual acuity^3^.

Variation in visual acuity has been tested for an association with neurodevelopmental conditions such as ADHD and autism spectrum disorder (ASD) with varied results^4–7^. The magnocellular theory of dyslexia suggests that a large part of the variance in reading skill is determined by visual and auditory sensitivity^8^. Several studies have focused on possible associations between visual acuity and reading skill. Among 1910 US school children, students with poor visual acuity were equally likely to be above-average, average or below-average readers^9^. In contrast, in a UK sample (overall *n* = 9545), children with bilateral deficits in near visual acuity (but not children with, e.g., unilateral or distance visual acuity deficits) were underachieving at reading after adjustment for socioeconomic status (SES), intelligence, and sex^10^. Moreover, children with dyslexia (*n* = 86) have been found to perform worse on near and distance visual acuity tests than sex-, age-, and IQ-matched controls^11^. Variation in visual acuity has been associated with early literacy at 4-5 years (*n* = 2025)^12^ but has not been found to be associated with school grades after adjusting for sex, ethnicity, school, reading skill, IQ, and paternal education^13^ suggesting that its proximal effects are on reading skill.

Understanding genetic factors contributing to visual function could help to dissect its links with neurodevelopmental conditions and reading skill. Several genome-wide association studies (GWAS) have been published on refractive error as a quantitative phenotype (*n* ≤ 542,934)^14–20^ or myopia in individuals of Asian (*n* ≤ 5030)^21–27^ and European descent (*n* ≤ 191,843)^28–31^. These GWAS identified up to 449 discrete loci (*n* = 542,934)^15^.

**Fig. 1:**
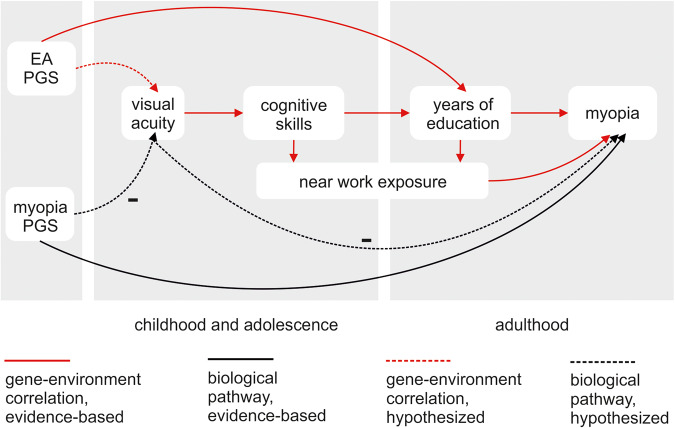
Associations between visual acuity, cognitive skills, and adult myopia. There is a negative link between visual acuity and myopia risk in childhood (not shown). Therefore, we expect that genetic factors known to increase myopia risk (myopia PGS) are negatively associated with visual acuity (black dotted arrow). However, visual acuity has been associated with increased cognitive skills, contributing to more years spent in education, which have been shown to increase myopia risk (red solid arrows). Therefore, we expect a positive association between the PGS for educational attainment (EA) and childhood visual acuity (red dotted arrow). The discrepancy of EA PGS being associated positively with childhood visual function and EA being associated negatively with adult visual function (in that it increases myopia risk) suggests gene-environment correlation. Specifically, the same factors associated with better vision earlier (EA PGS) are also associated with environmental exposures with detrimental effects on vision. Arrows represent a positive association unless otherwise indicated with a minus symbol (-).

Previous GWAS on visual function have almost exclusively been performed in older cohorts. However, the 39 lead single nucleotide polymorphisms (SNPs) associated with refractive error^28^ have shown different effects on refractive error in different age groups^32,33^ most likely due to gene-environment interactions^16^. For example, individuals at high genetic risk for myopia have a much more increased myopia risk if they have a University degree (OR ~ 51 compared to the general population) than if they only have primary schooling (OR ~ 7)^34^. Using Mendelian randomisation, it has been shown that higher educational attainment (EA) poses a causal risk factor for myopia (i.e., reduced visual function) rather than vice versa^35^. However, associations between visual acuity and cognitive measures described above suggest that visual and cognitive function are linked positively in childhood. This differing pattern in childhood vs. adulthood could be explained by gene-environment correlation. That is to say, the genetic background contributing to adult EA is associated with better childhood visual acuity (given its positive effect on cognitive skills), resulting in better school performance. This might lead to more time spent indoors, reading, and screen time and eventually increased myopia risk. If this is the case, polygenic scores (PGS) for EA should show a positive rather than negative association with childhood visual acuity (Fig. 1, red dotted arrow). Instead, a PGS for myopia represents a biological mechanism affecting visual function unrelated to the environment and should therefore show a negative association with childhood visual acuity (Fig. 1, black dotted arrow).

In the present study, we examined associations between visual acuity at the age of 11.8 years and cognitive measures including reading skill and school grades in the Avon Longitudinal Study of Parents and Children (ALSPAC) cohort (*n* = 6807). We performed a GWAS on visual acuity in children (*n* = 5571) and tested for associations with various cognitive measures, neurodevelopmental conditions, and visual phenotypes using PGS and genetic correlation analyses.

## Results

### Visual acuity and cognitive abilities

**Fig. 2:**
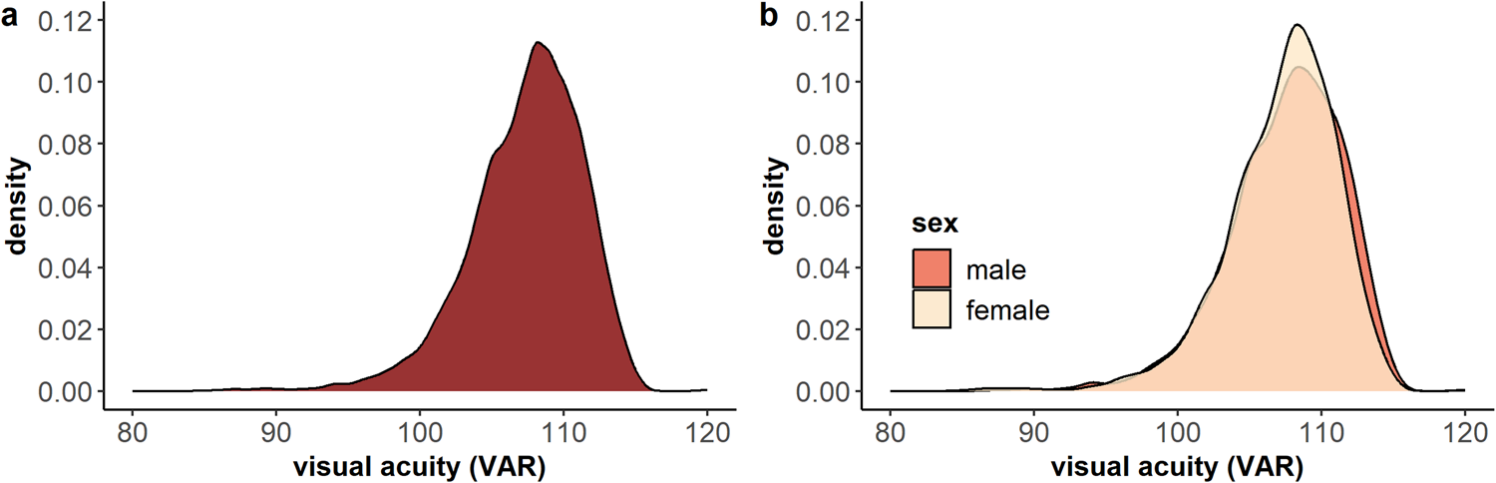
Distribution of visual acuity. **a** Distribution of visual acuity (better eye) in the overall sample (*n* = 6807), and **b** as a function of sex (n_females_ = 3444; n_males_ = 3356).

Visual acuity ranged from 85 to 120 (M = 107.38, SD = 3.93) (Fig. 2a; GWAS subset shown in Supplementary Figure 1). Boys showed slightly higher visual acuity (M = 107.52, SD = 3.97) than girls (M = 107.24, SD = 3.89), *t*_(6782.2)_ = 2.90, 95% CI = [0.08, 0.46], d = 0.07, *p* = .004 (Fig. 2b). Linear regression revealed a positive effect of age on visual acuity (*F*_(1,6805)_ = 20.44, ß = 0.02, SE = 0.004, adjusted R^2^ = 0.28, *p* = 6.3 × 10^−6^).

**Fig. 3:**
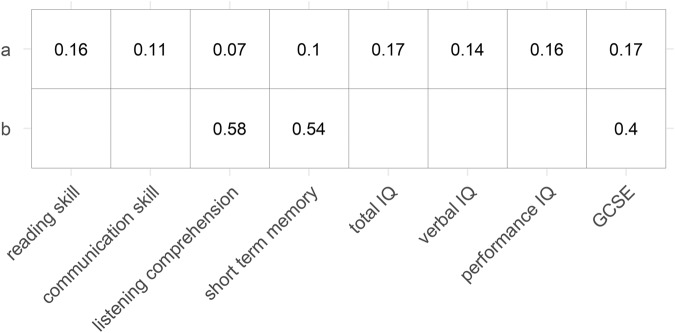
Correlation coefficients for visual acuity and cognitive measures. **a** Behavioural correlations. **b** Genetic correlations in unrelated individuals. Correlations are shown if passing the Bonferroni-corrected significance level (0.05/8 = 0.00625).

Partial correlation analyses adjusting for sex and age revealed positive correlations between visual acuity and cognitive performance including General Certificate of Secondary Education (GCSE) scores (all *p* < 3.0 × 10^−^^7^) (Fig. 3a, correlation plots in Supplementary Fig. 2). Partial correlation analysis between visual acuity and GCSE adjusting for sex, age, reading skill, verbal IQ, performance IQ, and SES revealed an attenuated, but significant association (*r*_(5662)_ = 0.05, 95% CI = [0.02, 0.08], *p* = 0.001).

We previously also reported associations between hearing ability and cognitive and neurodevelopmental traits^36^. To ensure that associations between visual acuity and cognitive traits are not driven by associations of visual acuity with hearing ability, we performed bivariate Pearson correlations on visual acuity and hearing threshold in the sample overlapping our previous study (*n* = 4929). Partial Pearson correlation analysis adjusting for sex and age did not reveal evidence for a correlation between visual acuity and hearing threshold (*r*_(4927)_ = −0.02, 95% CI = [−0.05, −0.005], *p* = 0.104), suggesting independence of hearing and vision phenotypes.

**Fig. 4:**
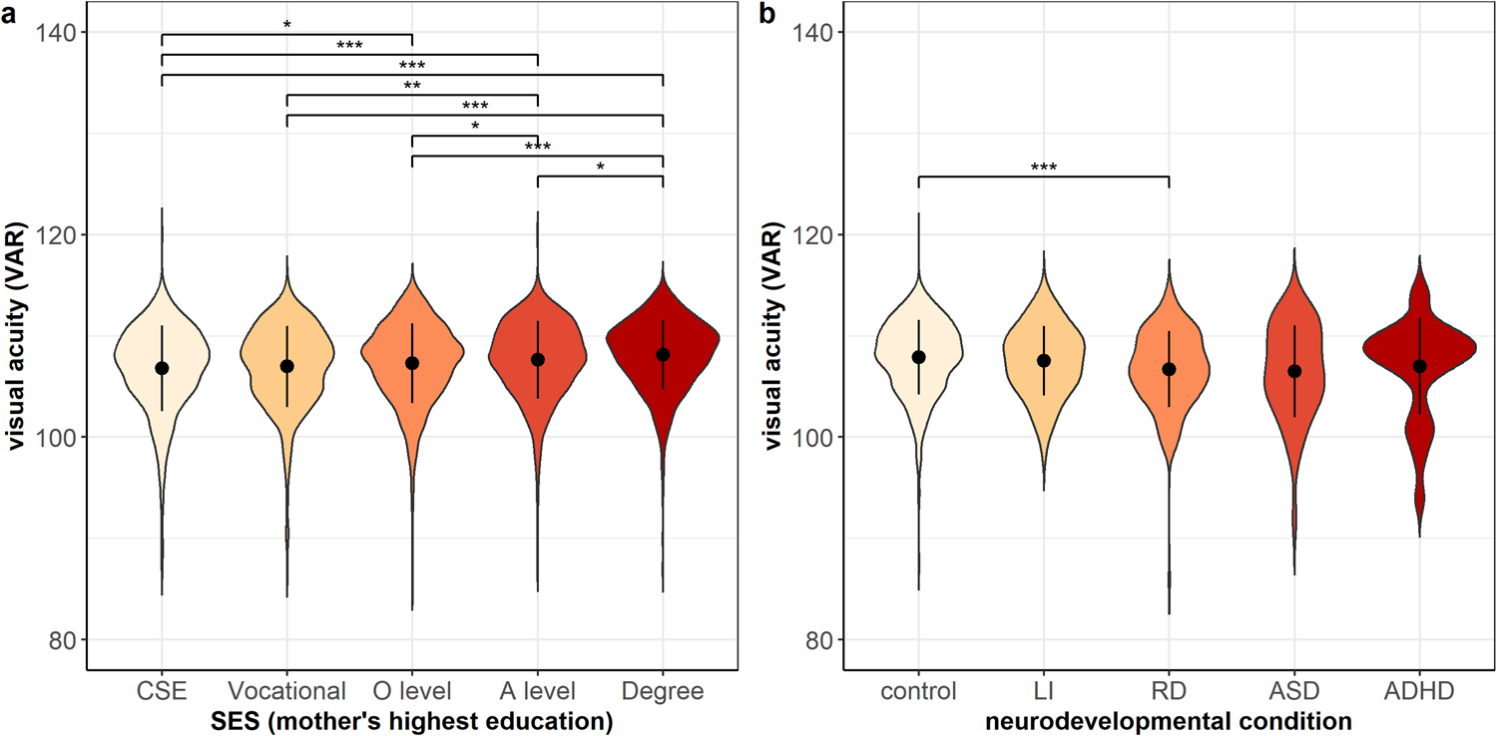
Visual acuity subgroup analyses. **a** grouped by SES and **b** grouped by neurodevelopmental conditions. Error bars of violin plots represent standard deviations (s.d.). ^***^p < 0 .001, ^**^p < 0.01, ^*^p < 0.05 in Tukey post-hoc tests after adjustment for multiple comparisons.

One-way between-subjects ANOVA revealed a significant main effect of SES on visual acuity after adjusting for sex and age (*F*_(4,6230)_ = 17.78, η^2^ = 0.01, 95% CI = [0.01, 1.00], *p* = 1.6 × 10^−^^14^). Post-hoc comparisons using the Tukey test indicated significant differences between most SES groups (Supplementary Tables 3 and 4), suggesting that higher SES is associated with better visual acuity (Fig. 4a). Moreover, there was a significant but small main effect of neurodevelopmental condition on visual acuity after adjusting for sex and age (*F*_(4,2666)_ = 6.54, η^2^ = 0.01, 95% CI = [0.00, 1.00], *p* = 3.1 × 10^−^^5^) with post-hoc comparisons indicating reduced visual acuity in the reading difficulty group (*n* = 203, mean = 106.71, SD = 3.77) compared to the control group (*n* = 2238, mean = 107.89, SD = 3.69, adjusted *p* = 0.0001) (Fig. 4b).

To ensure that the effect of neurodevelopmental group on visual acuity was not driven by outliers, the analysis was repeated after excluding individuals with visual acuity smaller than 3 standard deviations below the mean (16 individuals from the control group and one individual each from the reading difficulty, the language impairment, and the ADHD group). The main effect of neurodevelopmental condition on visual acuity remained significant before (*F*_(4,2649)_ = 6.71, η^2^ = 0.01, 95% CI = [0.00, 1.00], *p* = 2.3 × 10^−^^5^) and after adjusting for sex and age (*F*_(4,2647)_ = 6.75, η^2^ = 0.01, 95% CI = [0.00, 1.00], *p* = 2.1 × 10^−^^5^). Post-hoc comparisons for the unadjusted ANOVA show that the effect of reduced visual acuity in reading difficulty (*n* = 202, mean = 106.81, SD = 3.48) compared to controls (*n* = 2222, mean = 107.99, SD = 3.47, adjusted *p* = 4.6 × 10^−^^5^) was stronger after excluding outliers.

### Within sample genetic correlation

SNP-h^2^ for visual acuity (estimated using GCTA-GREML) was 0.26 (SE = 0.07, *p* = 4.5 × 10^−5^). Significant positive genetic correlation was found with listening comprehension, short term memory, and GCSE scores (Fig. 3b, Supplementary Table 5). There was no significant genetic correlation between visual acuity and hearing threshold (*n* = 3833, r_g_ = −0.56, SE = 0.73, *p* = 0.181).

### GWAS

**Fig. 5:**
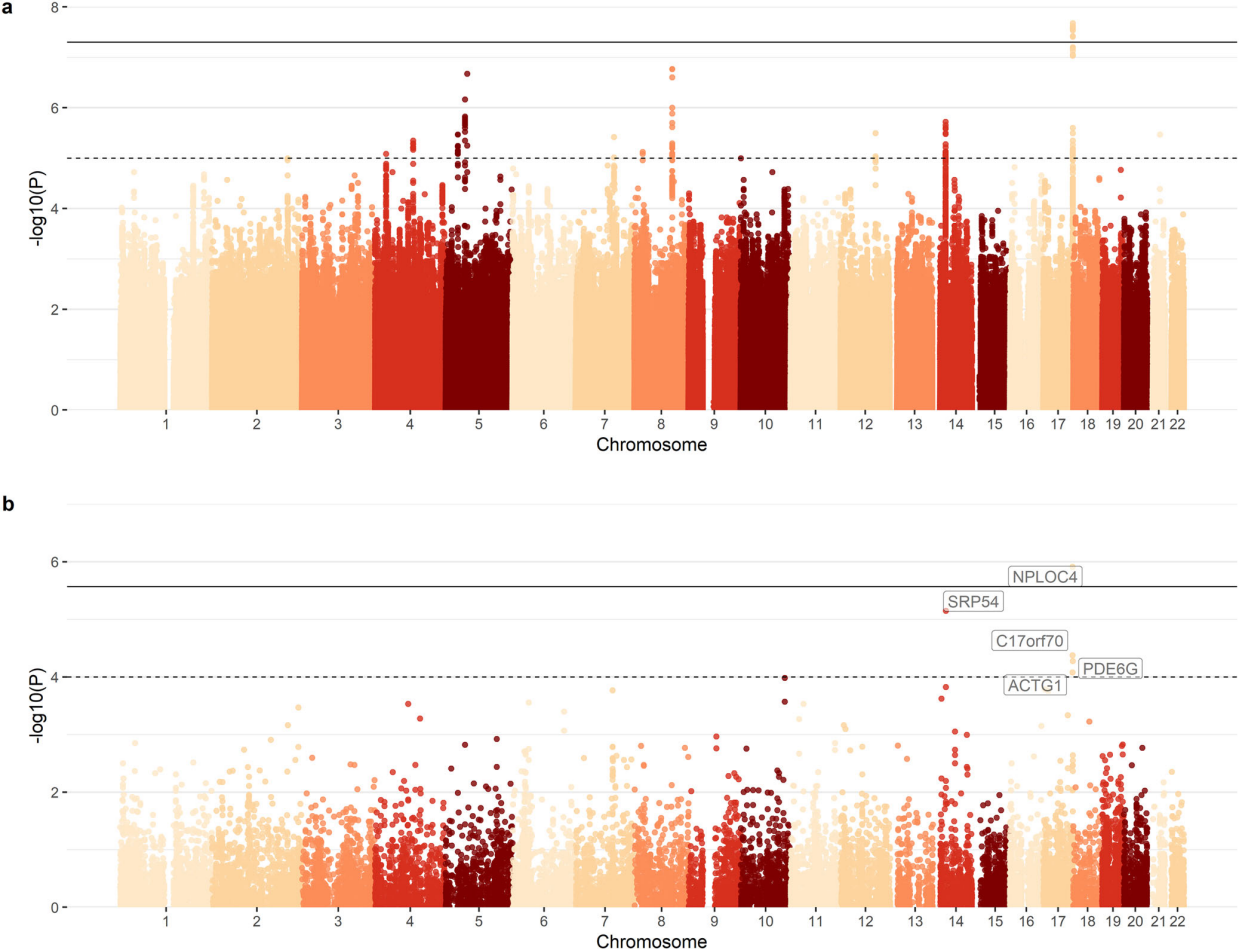
Manhattan plots for visual acuity. **a** SNP-based and **b** gene-based association *p* values are plotted against chromosome and position. SNP-based *p* values origin from linear mixed models in BOLT-LMM. Gene-based *p* values origin from re-weighting of SNP-based *p* values in MAGMA. The solid line represents the genome-wide significance level (**a**: *p* = 5 × 10^−^^8^, **b**: *p* = 2.7 × 10^−^^6^), the dotted line represents a suggestive significance level (**a**: *p* = 1 × 10^−^^5^, **b**: *p* = 1 × 10^−^^4^), respectively.

Twelve SNPs at the *NPLOC4* locus on chromosome 17 reached genome-wide significance (Fig. 5a, Supplementary Table 6, QQ plot Supplementary Fig. 3). The strongest association was found for rs11656126 (*p* = 2.1 × 10^−8^) with each copy of the major allele (G) (MAF = 0.35) shifting an individual 0.11 SD towards better visual acuity. The top marker on chromosome 5 (rs159195) is located in an intron of *PDE4D*.

In the targeted replication analysis of SNPs derived from previous literature, two genomic loci reached Bonferroni-corrected significance (Supplementary Table 7). The first locus spanned 20 SNPs in the chromosomal region 17:79526821-17:79630941, including the *NPLOC4* and the *PDE6G* gene. Among the 20 SNPs was rs6420484, for which each copy of the A allele has previously been associated with reduced refractive error (ß = −5.94, *p* = 2.8 × 10^−^^9^)^17^. Likewise, in our study, each copy of the A allele shifted an individual towards reduced visual acuity (MAF = 0.35, ß = −0.11, *p* = 9.3 × 10^−^^8^). The second locus spanned four SNPs in the chromosomal region 2:233372766-2:233385025, located in an intergenic region upstream of *PRSS56* previously associated with myopia^28^ and refractive error^16^.

### Post GWAS analysis

In line with SNP-based analysis, *NPLOC4* was the top hit in gene-based analysis (Fig. 5b, QQ plot Supplementary Fig. 4, *p* = 1.2 × 10^−^^6^). FUMA revealed that SNPs in the *NPLOC4* locus have been previously linked to sensory phenotypes such as advanced age-related macular degeneration, spherical equivalent (i.e., refractive error), and refractive astigmatism (GWAS catalog, Supplementary Table 8). The significant locus spans multi-tissue eQTLS influencing the expression of genes at the *NPLOC4* locus including *PDE6G* and *TSPAN10* (Supplementary Table 9). FINDOR re-weighted *p* values resulted in an uplift of the signals at chromosome 17 and 14 (Supplementary Fig. 5, Supplementary Table 10). In gene set enrichment analysis, “sensory perception of taste” (GO:0050909, *p* = 3.2 × 10^−^^6^) reached the Bonferroni-corrected significance level.

### Across samples genetic correlation

SNP-h^2^ for visual acuity (estimated using LDSC) was 0.18 (SE = 0.09). Visual acuity showed significant genetic correlations with cognitive performance and EA, but no genetic correlation with visual phenotypes in the UK Biobank (Table 1). Of note, smaller logMAR values correspond to better visual acuity, so negative correlation coefficients are expected. Sample sizes for visual phenotypes in UK Biobank are comparably small, explaining the large standard errors. It is of note that SNP-h^2^ for logMAR values in the UK Biobank are considerably smaller (0.03 and 0.02 for the left and right eye, respectively) than for ALSPAC children (0.18), suggesting that, in spite of a much smaller sample, analysis in individuals at young age might facilitate the detection of genetic effects.

**Table 1:** Across samples genetic correlation (LD Score regression) of visual acuity with cognitive and visual traits.

Trait	*n*	SNP-h^2^	r_g_ with visual acuity	SE	*p*
*Cognitive performance*^*a*^	**257,841**	**0.20**	**0.32**	**0.11**	**0.005**
*Educational attainment*^*a*^	**766,000**	**0.11**	**0.25**	**0.09**	**0.006**
logMAR (left)	79,239	0.03	−0.68	0.35	0.052
logMAR (right)	79,293	0.02	−0.34	0.31	0.281
Spherical power (left)	77,739	0.27	−0.04	0.10	0.699
Spherical power (right)	77,983	0.27	−0.03	0.11	0.769
Cylindrical power (left)	77,739	0.03	−0.34	0.25	0.183
Cylindrical power (right)	77,983	0.03	−0.25	0.23	0.291

^a^significant after Bonferroni correction for eight comparisons (*p* = 0.00625)

### PGS

EA PGS showed a robust positive association with visual acuity (Table 2) across multiple *p* value thresholds (Supplementary Fig. 6, Supplementary Table 11), indicating that genetic propensity towards higher EA is associated with better visual acuity in childhood. The standardised beta coefficient indicates that an increase of 1 SD in EA PGS is associated with an increase of 0.05 SD in visual acuity. This association still holds after including myopia PGS, refractive error PGS, or intelligence PGS as additional covariates to the PGS analysis (Supplementary Table 12). In contrast, myopia PGS showed a negative association with visual acuity with 1 SD increase in myopia PGS associated with a 0.05 SD decrease in visual acuity (Table 2). There was no association with PGS for neurodevelopmental outcomes.

**Table 2:** Results of PGS analysis on visual acuity (linear regression analyses with visual acuity as the outcome, PGS and covariates as predictors).

			Unstandardised		Standardised				
Trait	*p* value threshold^b^	*n* included SNPs	ß	SE	ß	SE	PGS R^2^ (%)	Full R^2^ (%)	*p*
ADHD	0.0036	3105	−86.60	39.83	−0.03	0.01	0.09%	2.01%	0.0297
Autism spectrum disorder	0.0390	13397	150.33	99.95	0.02	0.01	0.04%	1.96%	0.1326
Bipolar disorder	0.2306	52725	381.60	305.29	0.02	0.01	0.03%	1.95%	0.2114
Dyslexia	0.0002	1296	−124.84	40.19	−0.04	0.01	0.18%	2.10%	0.0019
Schizophrenia	5 × 10^-8^	349	8.56	4.84	0.03	0.01	0.06%	1.98%	0.0772
*Educational attainment*^*a*^	**0.0276**	**26404**	**2723.68**	**713.55**	**0.05**	**0.01**	**0.28%**	**2.20%**	**0.0001**
Intelligence	0.0074	11381	769.36	310.68	0.03	0.01	0.12%	2.04%	0.0133
*Myopia*^a^	**0.0062**	**6072**	−**359.92**	**97.43**	−**0.05**	**0.01**	**0.26%**	**2.18%**	**0.0002**
Refractive error	5 × 10^-8^	648	0.19	0.11	0.02	0.01	0.06%	1.98%	0.0790

^a^significant after Bonferroni correction (*p* = 0.00062) and indicated in bold.

^b^*p* value threshold with the highest predictive power for visual acuity. For other tested *p* value thresholds, see Supplementary Table 11.

## Discussion

We report behavioural and genetic associations of visual acuity with cognitive measures including reading skill and school grades in children. We found that better visual acuity is associated with higher SES, higher IQ scores, and increased language-related cognitive skills. Increased visual acuity at 11.8 years is associated with higher GCSE scores after adjusting for sex, reading skill, IQ, and SES, suggesting long-lasting effects of visual acuity on academic achievement.

Given the association with language-related skills, we investigated visual acuity in language-related neurodevelopmental conditions and found an association specifically with reading difficulty with behavioural data, in line with previous research^12^. According to the magnocellular theory dyslexia may result from the destabilisation of visual fixation^37^. The association between visual acuity and reading abilities was also observed in the overall sample regardless of a reading difficulty diagnosis. We did not find associations between PGS for neurodevelopmental disorders and visual acuity when controlling for SES. However, higher EA PGS were associated with better visual acuity in children before the end of compulsory education. These findings are consistent with theories that emphasise the importance of the senses in higher order cognition^38^.

Overall, 26% of the variance in visual acuity was attributed to genetic variation as captured by a genetic relationship matrix. The 12 SNPs reaching genome-wide significance in visual acuity GWAS were annotated to *NPLOC4*, which has been implicated in several GWAS on visual phenotypes previously. Gene-based analysis confirmed the *NPLOC4* association. The top marker on chromosome 5 (rs159195) is located in an intron of *PDE4D*, which shows abundant expression in the rodent retina^39^ and common variants in *PDE4D* have been reported in GWAS on sudden sensorineural hearing loss^40^. Targeted analysis for markers reported in previous GWAS for myopia and refractive error highlighted two loci in *NPLOC4* and *PDE6G*. *PDE6G* is expressed in photoreceptors and encodes for key enzymes of visual phototransduction signalling in the retina^41^.

Despite the small sample size, we both identified associations with genes involved in sensory function and replicated top hits from previous vision GWAS in adults. The direction of genetic correlation coefficients between logMAR values in the UK Biobank (r_g_ = −0.68 and −0.34 for the left and right eye, respectively) further suggests that our analysis captured similar genetics as previous relevant GWAS. The LDSC SNP-h^2^ estimate in our study (SNP-h^2^ = 0.18) is considerably higher than the ones in UK Biobank (0.03 and 0.02 for the same phenotype) despite a much smaller sample size. This indicates, consistent with our model, that over the course of development, environmental factors play increasingly larger effects on visual function, resulting in less phenotypic variance explained by genetic factors.

Since both EA PGS^42^ and access to eye examinations^43^ are associated with higher SES, we ran PGS analyses while controlling for SES. EA PGS showed a positive association with visual acuity, suggesting that the behavioural associations between visual acuity and cognitive skills are either mediated by shared biological pathways or can be explained by causal relationships. By including maternal education (used as a proxy measure for SES) as a covariate, we confirm that the EA PGS is associated with visual function after accounting for differential environments relevant to education provided by the mothers. However, we cannot rule out residual confounding by unmeasured variables.

In adults, it has been shown that a higher number of years spent in education is a causal risk factor for myopia^35^. Since low visual acuity is also a risk factor for myopia^3^ the positive effect of the EA PGS on childhood visual acuity is unexpected at first glance. However, as the children participating in this study have not yet reached the end of compulsory education, all have had the same number of educational years, suggesting that before environmental exposures are amplified by different educational trajectories, EA PGS are positively related to visual function. The association of higher EA with myopia in adulthood is thus likely to reflect gene-environment correlation. Specifically, genetic factors associated with higher EA might predispose to more reading and near work which increases myopia risk (Fig. 1). This is in line with a previous study confirming that EA PGS was not predictive for visual function in ALSPAC mothers (PGS R^2^ = 0.14%)^44^. In contrast, we found a less robust negative association between myopia PGS and childhood visual acuity and overall confirm the model introduced in Fig. 1. That being said, while our interpretation is that the effect of visual acuity on educational outcomes is mediated by cognitive abilities, we cannot exclude a reversed effect of education on cognition^45^.

A limitation of our study is that we used a single variable, i.e., maternal education, as a proxy measure for SES, rather than capturing other dimensions such as income-based SES measures. The maternal education measure has some advantages in that it is a better control of genetic confounding, that is, it is more heritable and more likely to be predictive of offspring achievement than an income-based measure. However, we acknowledge that it also has some limitations, namely, it may be less sensitive to confounding from wealth which might increase access to a stimulating learning environment and to healthcare such as optometry services and aids. Another limitation regarding the choice of phenotype is that vision and education have only been assessed at a single time point and the corrected visual acuity prevented us distinguishing the children wearing glasses or contact lenses during assessment or determine whether uncorrected refractive error was the underlying reason for reduced visual acuity. Future work will benefit from the use of longitudinal approaches and more distinguished visual phenotypes.

In summary, we confirm a positive association between childhood visual acuity and cognitive development. Specifically, we show that visual acuity is reduced in children with reading difficulty compared to controls. We conducted a GWAS for visual acuity and report a statistically significant association at the *NPLOC4* locus, implicated in vision phenotypes in previous studies conducted in larger adult cohorts. In the ALSPAC children, the PGS for educational attainment, which is known to be associated with myopia in adults, is instead associated with a slight increase in visual acuity. This observation is in line with the assumption that the initial positive effect of EA PGS on vision is reversed by environmental exposures, e.g., more near work exposure, increasing myopia risk.

## Methods

### Cohort

ALSPAC is a UK population-based longitudinal cohort. Pregnant women resident in the county of Avon, UK, with expected dates of delivery from 1st April 1991 to 31st December 1992 were invited to take part in the study, resulting in 14,062 live births and 13,988 children who were alive at 1 year of age^46,47^. When the oldest children were approximately 7 years of age, eligible cases who had failed to join the study originally were contacted again, resulting in an additional 913 children being enrolled. The total sample size for data collected after the age of seven is therefore 15,454 pregnancies, resulting in 15,589 foetuses. Of these 14,901 were alive at 1 year of age.

Informed written consent was obtained from the mother for analysis of her biological samples for genetic and other purposes, and by the accompanying parent for visual and other in person tests. Informed consent for the use of data collected via questionnaires and clinics was obtained from participants following the recommendations of the ALSPAC Ethics and Law Committee at the time. Parents were informed throughout that they, or their children, could withdraw at any time^48^. At age 18, study children were sent ‘fair processing’ materials describing ALSPAC’s intended use of their health and administrative records and were given clear means to consent or object via a written form. Data were not extracted for participants who objected, or who were not sent fair processing materials.

Ethical approval for the present study was obtained from the ALSPAC Law and Ethics Committee and the Local Research Ethics Committees. Details about the ethics committees and institutional review boards that approved aspects of the study are available on the ALSPAC study website (http://www.bristol.ac.uk/alspac/researchers/research-ethics/).

### Phenotypes

The ALSPAC study website contains details of all the data that is available through a fully searchable data dictionary (http://www.bris.ac.uk/alspac/researchers/data-access/data-dictionary/).

#### Assessment of visual acuity

Visual examinations were conducted in a shaded room under artificial light. Visual acuity was measured using the logarithm of the minimum angle of resolution (logMAR) visual acuity test with glasses or contact lenses if prescribed unless they had not been worn for at least six months. The order of testing was randomised between the left and right eye. The child was asked to read (or match) the first letter on each row and to go as far down the chart as possible. When they reached the point where they were unsure or made a mistake, they were taken back up two rows and asked to read across the row, letter by letter, with the examiner marking off each letter seen on the datasheet. If the child did not read the whole row correctly then the next row up was tested until there was one line fully correct. The child was then asked to continue reading down the chart. If the child read a letter incorrectly the examiner could check with a large letter whether the child knew the correct name for that letter, otherwise any errors were noted by not crossing off that letter. If one letter on a line was seen, all the rest were attempted. If a child said that they were not sure, they were encouraged to guess. Testing continued until they read a whole line wrong. The test was then repeated using the same chart but with the child holding up a pinhole. After a break used for another task, the ETDRS chart 2 was used for the second eye and the whole testing procedure repeated, including the pinhole. The number of letters in each column not seen, below the lowest fully correct line, were counted and visual acuity for each test (without and with pinhole) was calculated according to the formula: logMAR = −0.3 × (total errors × 0.02). For each eye, the best corrected visual acuity was determined as the best result from both tests (without and with pinhole). The logMAR value can adapt values from 1.00 (indicating poor vision) to −0.30 (indicating very good vision). We used the smallest value from the better eye as an indicator of visual acuity. To make the results more intuitive so that larger values refer to better visual acuity, we transformed logMAR to the Visual Acuity Rating (VAR) scale according to the formula visual acuity = 100–50(logMAR). Therefore, visual acuity = 100 refers to standard vision (logMAR = 0), while lower values indicate poorer vision and higher values indicate above-standard vision^49^. Phenotypic data were available for *n* = 6906 children. Children with sensory impairments (*n* = 26), visual acuity <85 (reduced visual acuity defined as visual impairment, *n* = 14^50^) or an absolute difference in visual acuity between the left and right eye of >10 (a possible indicator for amblyopia, *n* = 53^50^) were excluded from analysis, resulting in a sample of *n* = 6807 that was available for phenotypic analysis (3444 females, 3356 males, 7 missing values for sex, mean age = 11.81 years, SD = 0.23 years), of which ~13% had corrected vision.

#### Cognitive measures

In line with our previous study^36^ cognitive skills were assessed in terms of reading skill, communication skills, listening comprehension, short-term memory, total IQ, verbal IQ and performance (nonverbal) IQ, and EA measured as General Certificate of Secondary Education (GCSE) scores.

#### Reading skill

Reading skill was measured using the basic reading subtest of the Wechsler Objective Reading Dimensions (WORD)^51^ at target age 7. Seven pictures were used for decoding and 48 words were used for word reading. The task was ended after six consecutive errors. The final reading skill score was derived by the number of correctly read words and corrected for age in weeks.

#### Short term memory

Short term memory was measured using an adaptation of the Nonword Repetition Test^52^ at target age 8. The child was asked to listen to and repeat nonsense words presented via an audio cassette recorder. Twelve nonsense words (consisting of either 3, 4 or 5 syllables) conforming to English rules for sound combinations were used. Items were scored as correct if there was no phonological deviation from the presented word. The number of correctly repeated items was scored and corrected for age in weeks.

#### Listening comprehension

The Wechsler Objective Language Dimensions (WOLD)^53^ was used to measure listening comprehension at target age 8. The tester reads aloud a paragraph about a picture that is shown to the child. Afterwards, the child verbally answers questions about that paragraph that require making inferences about what they heard. The task was ended after three consecutive errors. A sum score was calculated from the number of correct items (ranging from 2–15) and corrected for age in weeks.

#### WISC performance IQ

The Wechsler Intelligence Scale for Children (WISC-III UK)^54^ was administered at target age 8. The WISC comprises five performance subtests (picture completion, coding, picture arrangement, block design, object assembly). Using the WISC manual, age-scaled scores were obtained from the raw scores and a total score was calculated for the WISC performance IQ.

#### WISC verbal IQ

The WISC includes five verbal subtests (information/knowledge, similarities, mental arithmetics, vocabulary, comprehension). Age-scaled scores were obtained as described above and a total score was calculated for the WISC verbal IQ.

#### WISC total IQ

The total WISC IQ score was calculated as the sum of all 10 age-scaled WISC subtests (picture completion, coding, picture arrangement, block design, object assembly, information/knowledge, similarities, mental arithmetics, vocabulary, comprehension).

#### Communication skills

Communication skills were measured using the children’s communication checklist (CCC)^55^ at target age 9. The CCC consists of 70 items grouped into 9 subscales (intelligibility and fluency, syntax, appropriate initiation, coherence, stereotyped conversation, use of conversational context, conversational rapport). Higher scores indicate higher communication skills. Sum scores were corrected for age in weeks.

#### GCSE scores

Educational attainment (EA) was measured as capped General Certificate of Secondary Education (GCSE) scores. GCSEs are the main qualification taken at the end of compulsory education in the UK. Capped GCSE scores represent the best eight grades at GCSE. GCSE scores were available for 6773 children.

#### SES

SES was assessed using maternal highest educational qualification during pregnancy (at 32 gestational weeks)^56^. SES was grouped into ‘CSE and no education’, ‘Vocational’, ‘O level’, ‘A level’ and ‘University Degree’.

#### Hearing

We previously reported associations between hearing ability and cognitive and neurodevelopmental traits^36^. The overlap between the sample used for the current study and the sample used for the previous study was *n* = 4929 (2490 females, 2439 males). Detailed descriptions of the hearing phenotypes have been reported previously^36^. Briefly, air conduction thresholds were assessed using an audiometer at different frequencies and averaged for each ear. Hearing threshold was defined as the average air conduction threshold in decibel (dB) on the better ear (note that lower values correspond to better hearing). We tested for correlations between visual acuity and hearing threshold using Pearson correlation.

### Subsample assignment

Group assignment followed the strategy described previously^57^. Briefly, from the overall ALSPAC sample (*n* = 15,443) we excluded individuals with incomplete data on measures used for sample assignment and individuals not reporting white European ethnicity. Next, individuals with a WISC performance IQ below 85 were excluded. The remaining individuals were assigned to the groups of reading difficulty (*n* = 173), language impairment (*n* = 184), ADHD (*n* = 26), comorbid combinations of these disorders (language impairment + reading difficulty, *n* = 47; language impairment+ADHD, *n* = 7, reading difficulty+ADHD, *n* = 5; reading difficulty+language impairment+ADHD, *n* = 3) or unaffected (*n* = 3305) according to the following criteria.

#### Reading difficulty

Children scoring < −1 SD on tests of age-adjusted single-word reading at 7 and 9 years were assigned to the reading difficulty group.

#### Language impairment

An assignment of language impairment was given if an individual scored positive for at least two of the following four criteria, which target different aspects of language problems:

1. CCC score < −1 SD
2. Nonword Repetition < −1 SD
3. WOLD score < −1 SD
4. positive response on speech/language therapy questionnaire

#### ASD

An assignment to ASD was based on maternal report (Have you ever been told that your child has autism, Asperger’s syndrome or autistic spectrum disorder?) at the age of 9 years.

#### ADHD

An assignment of ADHD was based on a DAWBA DSM-IV clinical diagnosis.

Numbers of participants were slightly different from previous publications given updates in the most recent release of the ALSPAC data.

The sample used in the present study (*n* = 6807) included 392 affected (language impairment, *n* = 159; reading difficulty, *n* = 155; language impairment + reading difficulty, *n* = 40, ADHD, *n* = 26, language impairment+ADHD, *n* = 4, reading difficulty+ADHD, *n* = 5, reading difficulty +language impairment+ADHD, *n* = 3) and 2953 unaffected individuals. Since we were specifically interested in reading difficulty, children with reading difficulty and comorbid disorders were assigned to the reading difficulty group (reading difficulty+language impairment, *n* = 40; reading difficulty+ADHD, *n* = 5; reading difficulty+language impairment+ADHD, *n* = 3), resulting in a reading difficulty subgroup of *n* = 203.

The control group was sex-matched to maintain the same M/F ration of 1.57 observed in the cases, resulting in a control sample matched for sex of *n* = 2238 (Table 3).

**Table 3:** Sample sizes of neurodevelopmental subgroups.

Subgroup	*n*	*n male*	*n* female	Ratio male: female
Unaffected	2238	1366	872	1.57
Affected	435	266	169	1.57
Language impairment	159	80	79	1.01
Reading difficulty	203	130	73	1.78
ASD	47	31	16	1.94
ADHD	26	25	<5	25.00

### Genomic analyses

Genome-wide genotype data were available for *n* = 5571 children with phenotypes (2749 males, 2822 females). Visual acuity was inverse rank-transformed for genomic analyses.

#### Genotype quality control and imputation

Genotypes were generated on the Illumina HumanHap550-quad array at the Wellcome Trust Sanger Institute, Cambridge, UK and the Laboratory Corporation of America, Burlington, NC, US. Standard quality control was performed as described elsewhere^58^. Population stratification was assessed by multidimensional scaling analysis and compared with Hapmap II (release 22) European descent, Han Chinese, Japanese and Yoruba reference populations. All individuals with non-European ancestry were removed. In total, 9115 subjects and 500,527 SNPs passed quality control filtering. Haplotypes were estimated using ShapeIT (v2.r644) which utilises relatedness during phasing. Quality control-filtered autosomal SNPs were imputed using Impute v3 using the HRC 1.1 reference data panel. Poorly imputed SNPs (Info score < 0.8) and SNPs with low minor allele frequency (MAF < 0.05) were excluded from further analysis. Overall, 5,305,352 SNPs with MAF > 0.05 that were either directly genotyped or imputed and passed quality control were tested for association with visual acuity.

#### SNP-h^2^ and genetic correlations

A genetic-relationship matrix was created for unrelated individuals (*n* = 5153; identity by descent (IBD) < 0.05) based on directly genotyped SNPs. SNP-h^2^ was estimated using sex, age in weeks, and the first two principal components (PCs) as covariates using GREML analysis in GCTA 1.94.0^59^. PCs were calculated using genotyped (MAF > 0.05) and LD pruned (r^2^ < 0.01 within a 50 kb window) SNPs (excluding high LD regions) using Plink v2^60^. We used bivariate GREML to estimate genetic correlations (r_g_) between visual acuity and cognitive skills. We also tested for genetic correlation between visual acuity and hearing threshold in *n* = 3833 unrelated children with genotypes and phenotypic data for both visual acuity and hearing threshold.

#### GWAS

Association testing for visual acuity was performed using a linear mixed model (LMM) in BOLT-LMM v2.3.4^61^. GWAS was run on the whole available sample with phenotypes and genotypes using sex, age, and the first two PCs (see above) as covariates (*n* = 5571).

#### Replication of previous studies

We tested for replication of SNPs associated with refractive error [*p* < 5 × 10^−8^^15–17,20^] or myopia [*p* < 5 × 10^−8^^28^] in previous large-scale GWAS. We selected the resulting 1,033 SNPs as pre-defined lead SNPs in FUMA, which identified 661 independent SNPs (r^2^ ≤ 0.6). Independent SNPs were used to determine boundaries of LD blocks that encompassed a total of 94 loci including 2,110 SNPs (r^2^ ≥ 0.6). Among these 2,110 SNPs, 743 (representing all 94 loci) were available in our study (Bonferroni-corrected significance level: 0.05/94 = 5.32 × 10^−^^4^).

#### LD score regression

We applied LD score regression (LDSC)^62^ implemented in the Complex Traits Genetics Virtual Lab (CTG-VL 0.4-beta)^63^ to the GWAS summary statistics for visual acuity. Mean χ^2^ was 1.03 and the small LDSC intercept (1.01, SE = 0.007) indicates negligible bias contributing to the signal. The genomic inflation factor revealed no evidence of population structure (λ = 1.05). Genetic correlation analyses^64^ were performed with traits of interest from publicly available GWAS summary statistics (Supplementary Table 1).

#### Post GWAS analysis

We applied FUMA v1.3.7^65^ and FINDOR^66^ on the GWAS summary statistics for visual acuity. FUMA implements MAGMA v1.08^67^ to perform gene-based analysis and gene set analysis. Functional consequences of SNPs were obtained by performing ANNOVAR^68^ using Ensembl genes (build 92). Functionally annotated SNPs were mapped to genes based on positional mapping. Intergenic SNPs were annotated to the closest genes upstream and downstream. Input SNPs were mapped to 18,360 protein-coding genes. Genome-wide significance for gene-based GWAS was defined as *p* = 0.05/18,360 = 2.7 × 10^−^^6^. Gene set *p* values were computed for 7343 gene ontology (GO) terms for biological processes obtained from MsigDB v5.2. The Bonferroni-corrected significance level was set to 0.05/7343 = 6.8 × 10^−^^6^. FUMA was also used to link independent significant SNPs to results from previous GWAS via the GWAS catalog. The visual acuity summary statistics were reweighted for functional annotations using FINDOR.

#### PGS

PGS analyses were carried out using PRSice 2.3.3^69^. We tested for associations of PGS for neurodevelopmental disorders (ASD, bipolar disorder, ADHD, schizophrenia, dyslexia), education (intelligence, EA), and visual function (refractive error, myopia) on visual acuity (Supplementary Table 2). After LD-clumping (r^2^ < 0.1 within a 250 kb window), PGS were derived as the weighted sum of risk alleles according to test statistics from the base GWAS summary statistics. We performed linear regression analyses with visual acuity as the outcome and the respective PGS as predictor. Based on an effect of SES on visual acuity on the behavioural level, SES, sex, age, and two PCs were included as covariates, resulting in a sample size of *n* = 5160 for PGS analyses. The Bonferroni-corrected significance threshold for nine base GWAS and nine *p* value thresholds (the threshold explaining maximal phenotypic variance as well as 0.001, 0.05, 0.1, 0.2, 0.3, 0.4, 0.5, and 1) was set to 0.05/81 = 0.00062.

Data preparation and visualization was performed using R v.4.1.2. All analysis scripts are available through Github (https://github.com/Judith-Schmitz/vision_gwas).

### Reporting summary

Further information on research design is available in the Nature Research Reporting Summary linked to this article.

## Supplementary information

Supplements

Reporting Summary

Supplementary_Tables

## Data Availability

Data used for this submission will be made available on request to the ALSPAC Executive (alspac-exec@bristol.ac.uk). The ALSPAC data management plan (http://www.bristol.ac.uk/alspac/researchers/data-access/documents/alspac-data-management-plan.pdf) describes the data sharing policy, which is through a system of managed open access.

## Code availability

All analysis scripts are available through Github (https://github.com/Judith-Schmitz/vision_gwas).
