# Supplementary material for: Identification of loci involved in childhood visual acuity and associations with cognitive skills and educational attainment
Source: NPJ Sci Learn. 2023 Jul 25;8:25. doi: 10.1038/s41539-023-00175-w (PMC10368730; doi:10.1038/s41539-023-00175-w)
Supplement: Supplementary file 1 — Supplements [file 41539_2023_175_MOESM1_ESM.pdf]

Supplementary Material:

Identification of loci involved in childhood visual acuity and associations with cognitive skills and educational attainment

Judith Schmitz, Filippo Abbondanza, Krzysztof Marianski, Michelle Luciano, Silvia Paracchini

Supplementary Figures

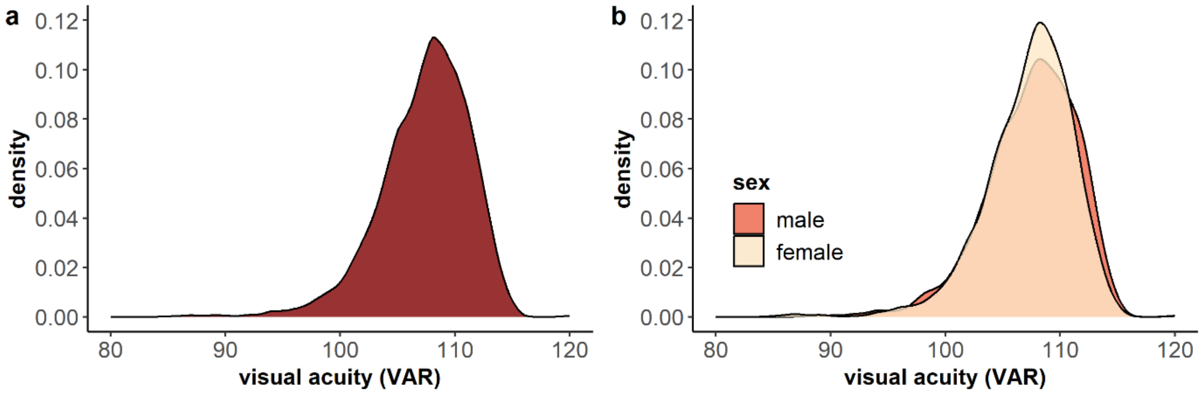

Supplementary Figure 1: Distribution of visual acuity in the GWAS subsample (n = 5,571). a) Distribution of visual acuity (better eye), and b) as a function of sex.

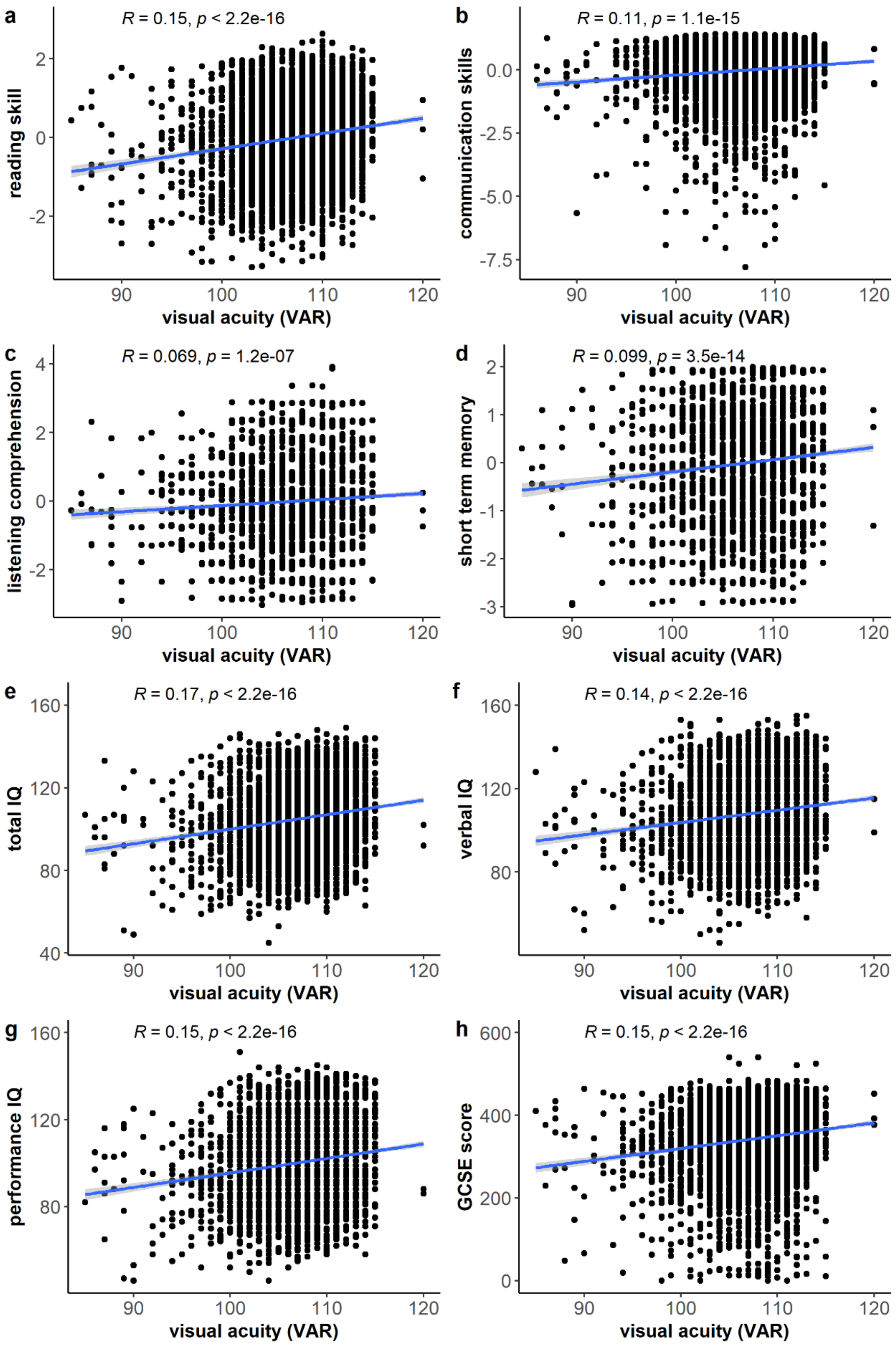

Supplementary Figure 2: Correlation plots between cognitive measures and visual acuity. All correlation coefficients are positive, indicating better visual acuity is associated with better cognitive skills. Correlation plots show unadjusted Pearson correlation coefficients, therefore correlation coefficients differ slightly from those in the main manuscript, which show partial correlation coefficients adjusting for sex and age.

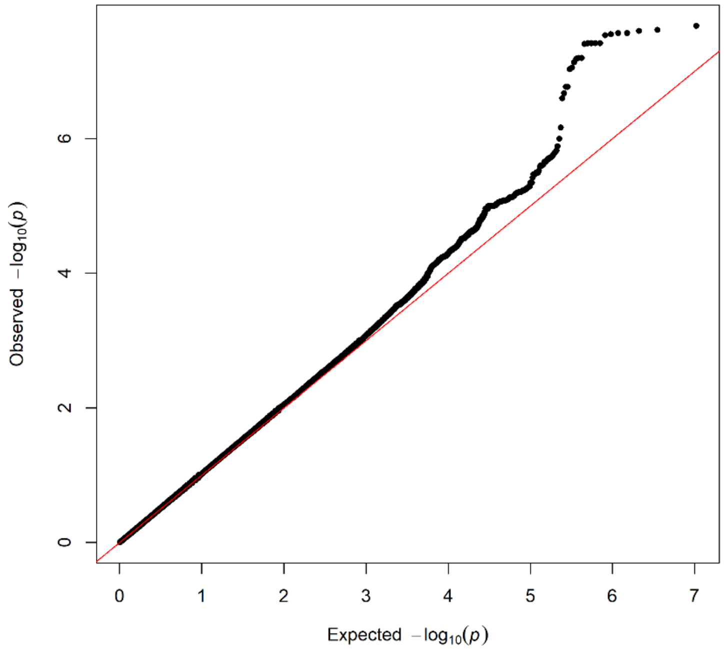

Supplementary Figure 3: QQ plot for GWAS on visual acuity. The observed SNP-based p values origin from linear mixed models in BOLT-LMM.

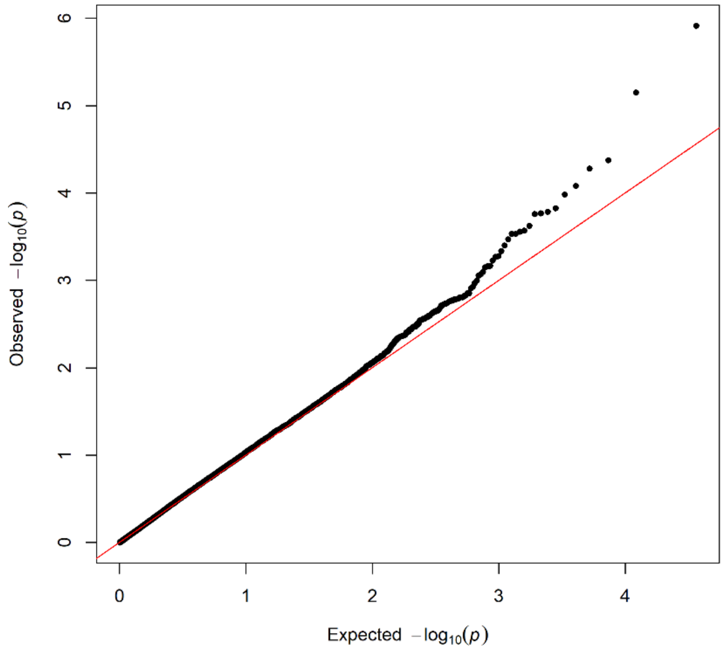

Supplementary Figure 4: QQ plot for gene-based analysis on visual acuity. The observed p values origin from a re-weighting of the BOLT-LMM SNP-based p values on the gene level performed using MAGMA.

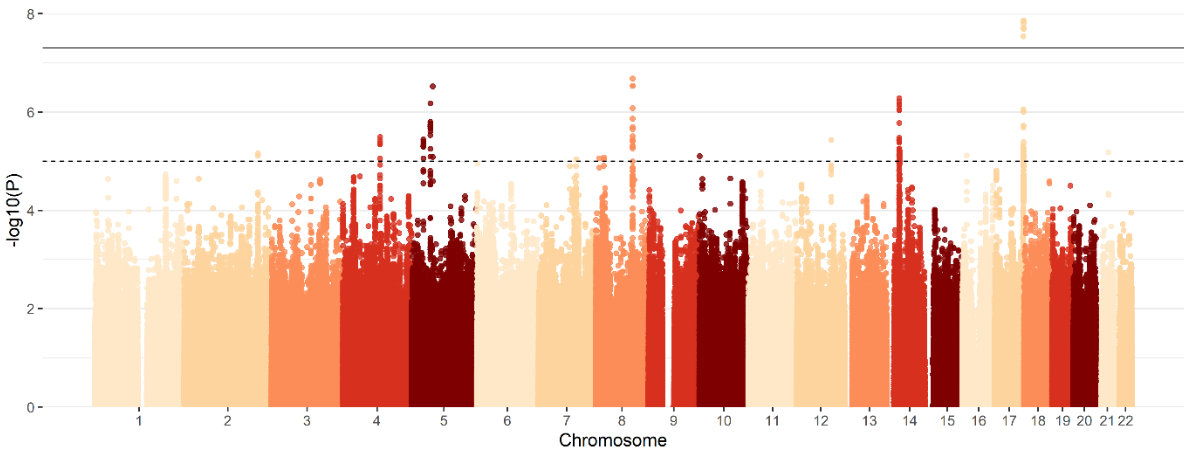

Supplementary Figure 5: FINDOR re-weighted Manhattan plot for visual acuity. p values (from re-weighting of the BOLT-LMM SNP-based p values for functional annotation in FINDOR) are plotted against chromosome and position. The solid line represents the genome-wide significance level ($p = 5 \times 10^{-8}$), the dotted line represents a suggestive significance level ($p = 1 \times 10^{-5}$), respectively.

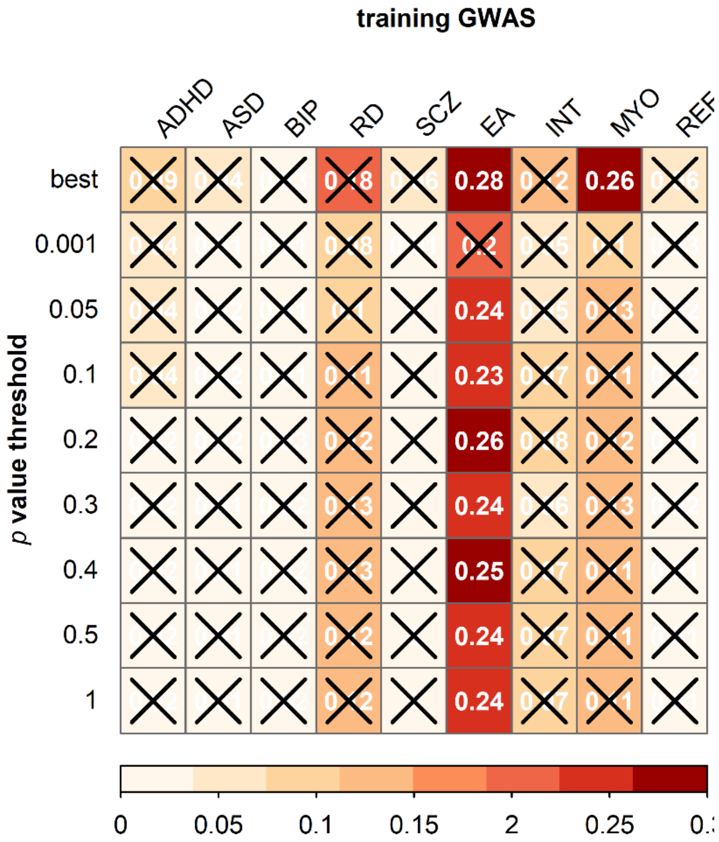

Supplementary Figure 6: PGS analysis on visual acuity. PGS R^2 (%) for linear regression analyses are reported for nine training GWAS and nine p value thresholds. R^2 not crossed out pass the Bonferroni-adjusted p value threshold. BIP = bipolar disorder, RD = reading difficulty, SCZ = schizophrenia, EA = educational attainment, INT = intelligence, MYO = myopia, REF = refractive error.

Supplementary Tables

Supplementary Table 1: Sample description for GWAS summary statistics used for LDSC genetic correlation analysis.

trait	cohort	reference	*n*	phenotype definition
logMAR (left)	UK Biobank	http://www.nealelab.is/uk-biobank/	79,239	Distance visual acuity in the left eye (wearing any prescribed optical correction)
logMAR (right)	UK Biobank	http://www.nealelab.is/uk-biobank/	79,293	Distance visual acuity in the right eye (wearing any prescribed optical correction)
Spherical power (left)	UK Biobank	http://www.nealelab.is/uk-biobank/	77,739	Spherical power of refractometry results
Spherical power (right)	UK Biobank	http://www.nealelab.is/uk-biobank/	77,983	Spherical power of refractometry results
Cylindrical power (left)	UK Biobank	http://www.nealelab.is/uk-biobank/	77,739	Cylindrical power of refractometry results
Cylindrical power (right)	UK Biobank	http://www.nealelab.is/uk-biobank/	77,983	Cylindrical power of refractometry results
Cognitive Performance	SSGAC	[1]	257,841	verbal-numerical reasoning or neuropsychological tests
Educational Attainment	SSGAC	[1]	766,000	highest level of education

Supplementary Table 2: Sample description for GWAS summary statistics used for PGS analysis.

trait	reference	download	*n*
ADHD	[2]	https://figshare.com/articles/dataset/adhd2019/14671965	19,099 cases, 34,194 controls
Autism spectrum disorder	[3]	https://figshare.com/articles/dataset/asd2019/14671989?file=28169292	18,382 cases, 27,969 controls
Bipolar disorder	[4]	https://figshare.com/articles/dataset/PGC3_bipolar_disorder_GWAS_summary_statistics/14102594	41,917 cases, 371,549 controls
Dyslexia[1]	[5]	https://research.23andme.com/collaborate/#dataset-access	51,800 cases, 1,087,070 controls
Schizophrenia	[6]	https://figshare.com/articles/dataset/scz2022/19426775	67,390 cases, 94,015 controls
Educational attainment	[7]	https://thessgac.com/papers/14	765,283
Intelligence	[8]	https://ctg.cncr.nl/software/summary_statistics	269,867
Myopia	[9]	https://research.23andme.com/collaborate/#dataset-access	85,757 cases, 106,086 controls
Refractive error	[10]	ftp://twinr-ftp.kcl.ac.uk/Refractive_Error_MetaAnalysis_2020	542,934

[1] The dyslexia summary statistics were not corrected for genomic control.

Supplementary Table 3: Descriptive results of visual acuity in SES groups.

**SES group**	***n***	**Mean visual acuity**	**SD visual acuity**
**CSE**	828	106.80	4.23
**Vocational**	530	106.98	4.01
**O level**	2,217	107.28	3.92
**A level**	1,664	107.64	3.81
**Degree**	998	108.14	3.45

Supplementary Table 4: Results of Tukey test for post-hoc analyses for unadjusted ANOVA of SES on visual acuity.

**Comparison**		**Difference**	**95%CI lower**	**95% CI upper**	p_{adj}
**Vocational**	**CSE**	0.17	-0.41	0.76	.930
**O level**	**CSE**	0.47	0.04	0.90	.022
**A level**	**CSE**	0.83	0.39	1.28	< .001
**Degree**	**CSE**	1.34	0.84	1.83	< .001
**O level**	**Vocational**	0.30	-0.21	0.81	.493
**A level**	**Vocational**	0.66	0.13	1.19	.006
**Degree**	**Vocational**	1.16	0.60	1.73	< .001
**A level**	**O level**	0.36	0.02	0.70	.033
**Degree**	**O level**	0.86	0.46	1.27	< .001
**Degree**	**A level**	0.50	0.08	0.93	.011

Supplementary Table 5: Within sample genetic correlation (bivariate linear mixed model, GREML analysis in GCTA) of visual acuity with cognitive traits.

**Trait**	***n***	r_g **with visual acuity**	**SE**	***p* value**
reading skill	4,529	.35	0.14	.010
communication skills	4,396	.58	0.27	.010
**listening comprehension***	**4,491**	**.58**	**0.17**	$\mathbf{2.0 \times 10^{-4}}$
**short term memory***	**4,484**	**.54**	**0.17**	$\mathbf{5.1 \times 10^{-4}}$
total IQ	4,455	.35	0.14	.008
verbal IQ	4,477	.30	0.14	.016
performance IQ	4,469	.35	0.16	.022
**GCSE***	**4,311**	**.40**	**0.14**	**.003**

** passing the Bonferroni-corrected significance level.*

Supplementary References

1. Lee, J. J. *et al.* Gene discovery and polygenic prediction from a genome-wide association study of educational attainment in 1.1 million individuals. *Nature genetics* **50,** 1112–1121 (2018).
2. Demontis, D. *et al.* Discovery of the first genome-wide significant risk loci for attention deficit/hyperactivity disorder. *Nature genetics* **51,** 63–75 (2019).
3. Grove, J. *et al.* Identification of common genetic risk variants for autism spectrum disorder. *Nature genetics* **51,** 431–444 (2019).
4. Mullins, N. *et al.* Genome-wide association study of more than 40,000 bipolar disorder cases provides new insights into the underlying biology. *Nature genetics* **53,** 817–829 (2021).
5. Doust, C. *et al.* Discovery of 42 genome-wide significant loci associated with dyslexia. *Nature genetics* **54,** 1621–1629 (2022).
6. Trubetskoy, V. *et al.* Mapping genomic loci implicates genes and synaptic biology in schizophrenia. *Nature* **604,** 502–508 (2022).
7. Okbay, A. *et al.* Polygenic prediction of educational attainment within and between families from genome-wide association analyses in 3 million individuals. *Nature genetics* **54,** 437–449 (2022).
8. Savage, J. E. *et al.* Genome-wide association meta-analysis in 269,867 individuals identifies new genetic and functional links to intelligence. *Nature genetics* **50,** 912–919 (2018).
9. Pickrell, J. K. *et al.* Detection and interpretation of shared genetic influences on 42 human traits. *Nature genetics* **48,** 709–717 (2016).
10. Hysi, P. G. *et al.* Meta-analysis of 542,934 subjects of European ancestry identifies new genes and mechanisms predisposing to refractive error and myopia. *Nature genetics* **52,** 401–407 (2020).
