# Supplementary material for: Identification of loci involved in childhood visual acuity and associations with cognitive skills and educational attainment
Source: NPJ Sci Learn. 2023 Jul 25;8:25. doi: 10.1038/s41539-023-00175-w (PMC10368730; doi:10.1038/s41539-023-00175-w)
Supplement: Supplementary file 2 — Reporting Summary [file 41539_2023_175_MOESM2_ESM.pdf]

nature portfolio

Corresponding author(s): Silvia Paracchini

Last updated by author(s): Jul 7, 2023

Reporting Summary

Nature Portfolio wishes to improve the reproducibility of the work that we publish. This form provides structure for consistency and transparency in reporting. For further information on Nature Portfolio policies, see our Editorial Policies and the Editorial Policy Checklist.

Statistics

For all statistical analyses, confirm that the following items are present in the figure legend, table legend, main text, or Methods section.

n/a	Confirmed	
☐	☒	The exact sample size (*n*) for each experimental group/condition, given as a discrete number and unit of measurement
☒	☐	A statement on whether measurements were taken from distinct samples or whether the same sample was measured repeatedly
☐	☒	The statistical test(s) used AND whether they are one- or two-sided<br>*Only common tests should be described solely by name; describe more complex techniques in the Methods section.*
☐	☒	A description of all covariates tested
☐	☒	A description of any assumptions or corrections, such as tests of normality and adjustment for multiple comparisons
☐	☒	A full description of the statistical parameters including central tendency (e.g. means) or other basic estimates (e.g. regression coefficient) AND variation (e.g. standard deviation) or associated estimates of uncertainty (e.g. confidence intervals)
☐	☒	For null hypothesis testing, the test statistic (e.g. *F*, *t*, *r*) with confidence intervals, effect sizes, degrees of freedom and *P* value noted<br>*Give P values as exact values whenever suitable.*
☒	☐	For Bayesian analysis, information on the choice of priors and Markov chain Monte Carlo settings
☒	☐	For hierarchical and complex designs, identification of the appropriate level for tests and full reporting of outcomes
☐	☒	Estimates of effect sizes (e.g. Cohen's *d*, Pearson's *r*), indicating how they were calculated

Our web collection on statistics for biologists contains articles on many of the points above.

Software and code

Policy information about availability of computer code

Data collection	No software was used for data collection.
Data analysis	R v.4.1.2 was used for data preparation, behavioural data analysis, and data visualization. Genomic analyses were performed using ShapeIT (v2.r644), Impute v3, GCTA v1.94.0, Plink v2, BOLT-LMM v2.3.4, LDSC implemented in the Complex Traits Genetics Virtual Lab (CTG-VL 0.4-beta), FUMA v1.3.7, FINDOR, and PRSice 2.3.3.

For manuscripts utilizing custom algorithms or software that are central to the research but not yet described in published literature, software must be made available to editors and reviewers. We strongly encourage code deposition in a community repository (e.g. GitHub). See the Nature Portfolio guidelines for submitting code & software for further information.

Data

Policy information about availability of data

All manuscripts must include a data availability statement. This statement should provide the following information, where applicable:

- Accession codes, unique identifiers, or web links for publicly available datasets
- A description of any restrictions on data availability
- For clinical datasets or third party data, please ensure that the statement adheres to our policy

Data used for this submission will be made available on request to the ALSPAC Executive (alspac-exec@bristol.ac.uk). The ALSPAC data management plan (http://

www.bristol.ac.uk/alspac/researchers/data-access/documents/alspac-data-management-plan.pdf) describes the data sharing policy, which is through a system of managed open access.

Human research participants

Policy information about studies involving human research participants and Sex and Gender in Research.

Reporting on sex and gender	The ALSPAC variable we use when we refer to "sex" is the biological sex at birth. We do not have information on gender, therefore we cannot provide numbers on diaggregated sex and gender. Sex has been included as a covariate in all analyses where appropriate.
Population characteristics	ALSPAC is a UK population-based longitudinal cohort of individuals born between 1991 and 1992.
Recruitment	Pregnant women resident in the county of Avon, UK, with expected dates of delivery from 1st April 1991 to 31st December 1992 were invited to take part in the study, resulting in 14,062 live births.
Ethics oversight	Ethical approval for the present study was obtained from the ALSPAC Law and Ethics Committee and the Local Research Ethics Committees.

Note that full information on the approval of the study protocol must also be provided in the manuscript.

Field-specific reporting

Please select the one below that is the best fit for your research. If you are not sure, read the appropriate sections before making your selection.

☒ Life sciences ☐ Behavioural & social sciences ☐ Ecological, evolutionary & environmental sciences

For a reference copy of the document with all sections, see nature.com/documents/nr-reporting-summary-flat.pdf

Life sciences study design

All studies must disclose on these points even when the disclosure is negative.

Sample size	Since the sample analysed in this study was pre-existing, we had no control over the sample size. Phenotypic data on visual acuity were available for 6,906 children (5,571 with available phenotypes).
Data exclusions	Children with sensory impairments (n = 26), VA < 85 (reduced visual acuity defined as visual impairment, n = 14) or an absolute difference in VA between the left and right eye of > 10 (a possible indicator for amblyopia, n = 53) were excluded from analysis, resulting in a sample of n = 6,807 that was available for phenotypic analysis. Cutoffs were chosen based on previous literature.
Replication	n/a
Randomization	n/a
Blinding	n/a

Reporting for specific materials, systems and methods

We require information from authors about some types of materials, experimental systems and methods used in many studies. Here, indicate whether each material, system or method listed is relevant to your study. If you are not sure if a list item applies to your research, read the appropriate section before selecting a response.

Materials & experimental systems

n/a	Involved in the study
☒	☐ Antibodies
☒	☐ Eukaryotic cell lines
☒	☐ Palaeontology and archaeology
☒	☐ Animals and other organisms
☒	☐ Clinical data
☒	☐ Dual use research of concern

Methods

n/a	Involved in the study
☒	☐ ChIP-seq
☒	☐ Flow cytometry
☒	☐ MRI-based neuroimaging
